# Veterinary education and experience shape beliefs about dog breeds. Part 2: Trust

**DOI:** 10.1038/s41598-023-40464-3

**Published:** 2023-08-24

**Authors:** Rachel M. P. Caddiell, Philip White, B. Duncan X. Lascelles, Kenneth Royal, Kimberly Ange-van Heugten, Margaret E. Gruen

**Affiliations:** 1 Comparative Behavioral Research, Department of Clinical Sciences, College of Veterinary Medicine, North Carolina State University, Raleigh, NC USA; 2 Department of Clinical Sciences, College of Veterinary Medicine, North Carolina State University, Raleigh, NC USA; 3 Department of Clinical Sciences, College of Veterinary Medicine, Translational Research in Pain, North Carolina State University, Raleigh, NC USA; 4 Department of Statistics, College of Physical and Mathematical Sciences, Brigham Young University, Provo, UT USA; 5 Comparative Pain Research and Education Center, College of Veterinary Medicine, North Carolina State University, Raleigh, NC USA; 6 Thurston Arthritis Centre, UNC School of Medicine, Chapel Hill, NC USA; 7 Department of Anesthesiology, Center for Translational Pain Research, Duke University, Durham, NC USA; 8 Department of Animal Science, College of Agriculture and Life Sciences, North Carolina State University, Raleigh, NC USA; 9 Environmental Medicine Consortium, North Carolina State University, Raleigh, NC USA

**Keywords:** Human behaviour, Health care

## Abstract

Dog breed stereotypes are frequently used to inform people’s expectations about canine behavior, despite evidence that breed is largely uninformative in predicting individual dog behavior. Further, these beliefs differ among populations. However, it remains unknown how ratings of warmth toward a breed are associated with ratings of other social behavioral domains, and whether differences exist between populations with varying experience with dogs. The purpose of this study was to evaluate ratings of trust and warmth among survey respondents including veterinary students, veterinary faculty and staff, undergraduates in animal-health related majors and members of the general public. Using an online survey, respondents rated their likelihood to trust a dog in varying scenarios for 10 different dog breeds. Additionally, respondents used a feelings thermometer to rate how warm or cool they felt towards each breed. Findings revealed differences in feelings thermometer and trust ratings across populations. All ratings were lower among the veterinary academic respondents compared to the general public and undergraduates. Veterinary students further along in their training, as well as undergraduates with clinical experience, reflected perceptions similar to those of the veterinary faculty and staff providing support for cultural transmission of beliefs during veterinary education and training.

## Introduction

Humans hold stereotypes about animal species that affect how they feel and behave toward them. As groups, animal species have been shown to be subject to several of the same mental processes that humans apply to groups of other humans, including empathic responses and ratings of warmth (positive perceived intent) and competence (high ability and capacity)^1,2^. These two dimensions, warmth and competence, are central to the stereotype content model first proposed by Fiske and colleagues to describe the social cognitive influences on how people form beliefs about group members^3,4^. At a species level, and within certain cultures, dogs (as a group) are associated with positive behavioral characteristics including friendliness^1^ and they rate high on both warmth and competence. In one survey study, respondents rated dogs highest in both warmth and competence, compared with 24 other animal species including cats, horses, elephants, and monkeys^2^.

However, as a social partner, dogs are also subject to stereotypes and discrimination *within* the species, with breed as the predominant driver of perceived differences in dog behavior and temperament^5–10^. Dog breed stereotypes about behavior are pervasive and shape people’s expectations about how an individual dog will behave in a given setting^7^. This is despite numerous studies demonstrating that variability within a breed is greater than among breeds^7,11,12^. While heritability for certain behavioral traits such as human sociability and biddability have been convincingly demonstrated^7,13,14^, breed is largely uninformative when it comes to predicting behavior in an individual dog^7^. Despite the lack of strong evidence for breed-based behaviors, breed and breed labeling remain central to decisions people make about dogs, including whether to adopt a dog from a shelter^6,10,15^.

Still, beliefs and stereotypes about dog breeds may be differentially expressed among populations. For example, beliefs held by the general public and by veterinarians are not always aligned. Surveys of veterinarians in the United States have found breed-based differences in perception of pain sensitivity^16^ and aggression (particularly as it relates to breed-specific legislation)^17^. In Gruen et al., veterinarians’ ratings of pain sensitivity for dogs of different breeds were significantly different from ratings made by general public members for the majority of the 28 dog breeds included in the survey^16^. Kogan et al. found that a majority (70%) of veterinarian respondents believed that some breeds of dogs are more likely to be aggressive than others and attributed the highest serious bite risk to chow chows, Chihuahuas, and German shepherds^17^. Pitbulls—a breed group or type rather than a specific breed—are often stigmatized and associated with high risk of aggression by the general public^8,15,18,19^; however, surveyed veterinarians rated them as moderate risk^17^. A similar pattern has been found cross-culturally, with small animal veterinarians in New Zealand rating pitbull type dog breeds as less aggressive than Chihuahuas, rottweilers, chow chows, German shepherds, and corgis^20^.

It is important to understand whether beliefs held by veterinarians differ from beliefs of the general public, and, if so, how these different beliefs develop as veterinarians play a major role in public health and medical treatment of dogs. In Kogan et al.’s 2019 survey of veterinarians, a majority agreed that veterinarians have a role in advising clients on how to train or manage aggressive or dangerous dogs^17^. Breed-based perceptions about aggression and other behavioral traits may influence how veterinarians advise their clients or handle patients in the clinic. Among human physicians, medical school training has been associated with the development (or maintenance) of stereotypes related to age, gender, and race, among others^21–23^; these beliefs have been shown to impact treatment, even in the absence of explicit bias^22^. We previously demonstrated that ratings of pain sensitivity in dogs of various breeds are affected by ratings of warmth toward a breed^16^. Among veterinarian responders, ratings of pain sensitivity were negatively correlated with ratings of warmth; this was the opposite finding for members of the general public. It is unknown how ratings of warmth toward a breed are associated with ratings for other social behavioral domains, and whether differences exist between populations, particularly those with veterinary medical training. The purpose of this study was to build upon previous findings to determine if population differences exist also for ratings of trust, warmth, and likelihood of adoption and expand the study by evaluating undergraduates in animal-centered majors, veterinary students at different levels of their training, and veterinary faculty and staff, compared to members of the general public. This would be the first demonstration of differences in these domains among these populations, and provide some insight into when and how these differences are formed. Based on findings from human medical training, we predicted that we would find differences among these populations, and that veterinary students, particularly in their later years of training, would more closely resemble veterinary faculty and staff than the general public or undergraduates. We further predicted that breed identification, through display of DNA composition in mixed breed dogs, would affect ratings in these populations. If found, these differences would lay the foundation for research on additional human populations, and underscore the importance of studying whether these differences impact treatment of dogs.

## Methods

This survey was conducted with members of the general public, undergraduates from North Carolina State University, and veterinary students, faculty, and staff at eight colleges of veterinary medicine in the United States (North Carolina State University, Auburn University, University of Georgia, Louisiana State University, Oregon State University, Tufts University, and Virginia Tech University). Data from one school (North Carolina State University) were collected from undergraduates as well as veterinary students, staff, and faculty; data were not collected from undergraduates at any other institution, but were collected for veterinary students, staff, and faculty. All responses were collected anonymously and written informed consent was obtained from all participants. All surveys were reviewed by North Carolina State University’s Institutional Review Board (Protocol #22285) and were performed in accordance with relevant guidelines and regulations; all participants were required to be over 18 years of age and reside in the United States. General public members were recruited using Amazon’s Mechanical Turk (mTurk) between October 25 and November 4, 2020; respondents were paid $0.75 for their participation. mTurk is a crowdsourcing website where workers are employed to complete on-line tasks including surveys. mTurk respondents had an identical survey to the other populations, with the exception of an additional attention check question embedded in the questionnaire; respondents who failed this attention check were excluded from analysis. This population of respondents has been employed in many online surveys, and attention checks have proven to be useful for ensuring the authenticity of responses^33,34^.

Undergraduate respondents were recruited from NC State University using listservs specific to students majoring in Animal Science, Zoology, and Biology. These were selected in order to recruit respondents with a high level of interest in and knowledge of animals, and are the majors with the highest proportion of pre-veterinary students. Veterinary students, faculty, and staff (including interns and residents) were recruited using listservs administered by each participating college of veterinary medicine. While invitations were sent to all veterinary colleges, responses were received from eight; participating colleges included: NC State University, Auburn University, University of Georgia, Iowa State University, Louisiana State University, Oregon State University, Tufts University, and Virginia Tech University; surveys remained open for an average of 3.5 weeks at each participating university. While responses were collected anonymously, interested student participants were invited to provide their email address so that they could be entered into a raffle to win one of 39 Amazon gift cards ($20 each).

**Figure 1:**
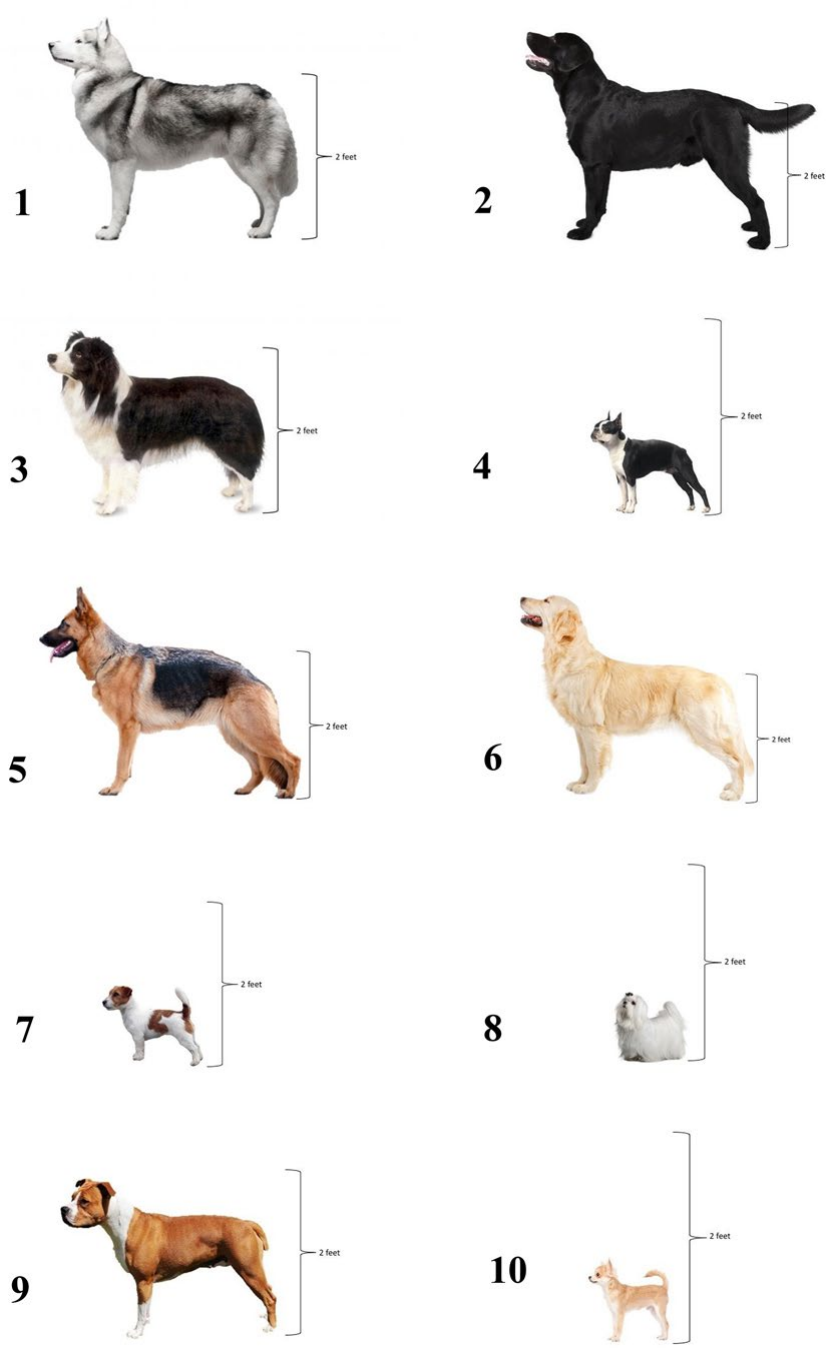
Standardized pictures presented to survey participants of ten dog breeds: (1) Siberian husky, (2) Labrador retriever, (3) border collie, (4) Boston terrier, (5) German shepherd, (6) golden retriever, (7) Jack Russell terrier, (8) Maltese, (9) pitbull type dog, and (10) Chihuahua.

The survey was adapted from Gruen et al.^16^ and developed using Qualtrics® survey software. Pilot testing was performed to ensure readability and feedback on question structure and disambiguation were incorporated into the final survey. The survey was structured in seven blocks. The first block included information about the survey and respondents gave informed consent before moving on to the second block. The second block included directions and an example for the picture and scale. The third block presented standardized pictures of ten different breeds of dogs (Siberian husky, Labrador retriever, border collie, Boston terrier, German shepherd, golden retriever, Jack Russel terrier, maltese, pitbull-type, and chihuahua) and six mixed breed dogs and asked respondents to rate their pain sensitivity on a scale from “Not at all sensitive” to “Most sensitive imaginable” (Fig. 1). Selection of these breeds and results from this block are discussed in the companion article (Caddiell, et al.). The fourth block presented the same ten purebred and six mixed-breed dogs but respondents were asked to rate the dogs on a scale from 0 = Not at all likely to 10 = Very likely for the variables: Trust this dog with young children; Adopt this dog into your house; Trust this dog with a cat or small animal; Trust this dog in a crowd of people; Take this dog to a park.

**Figure 2:**
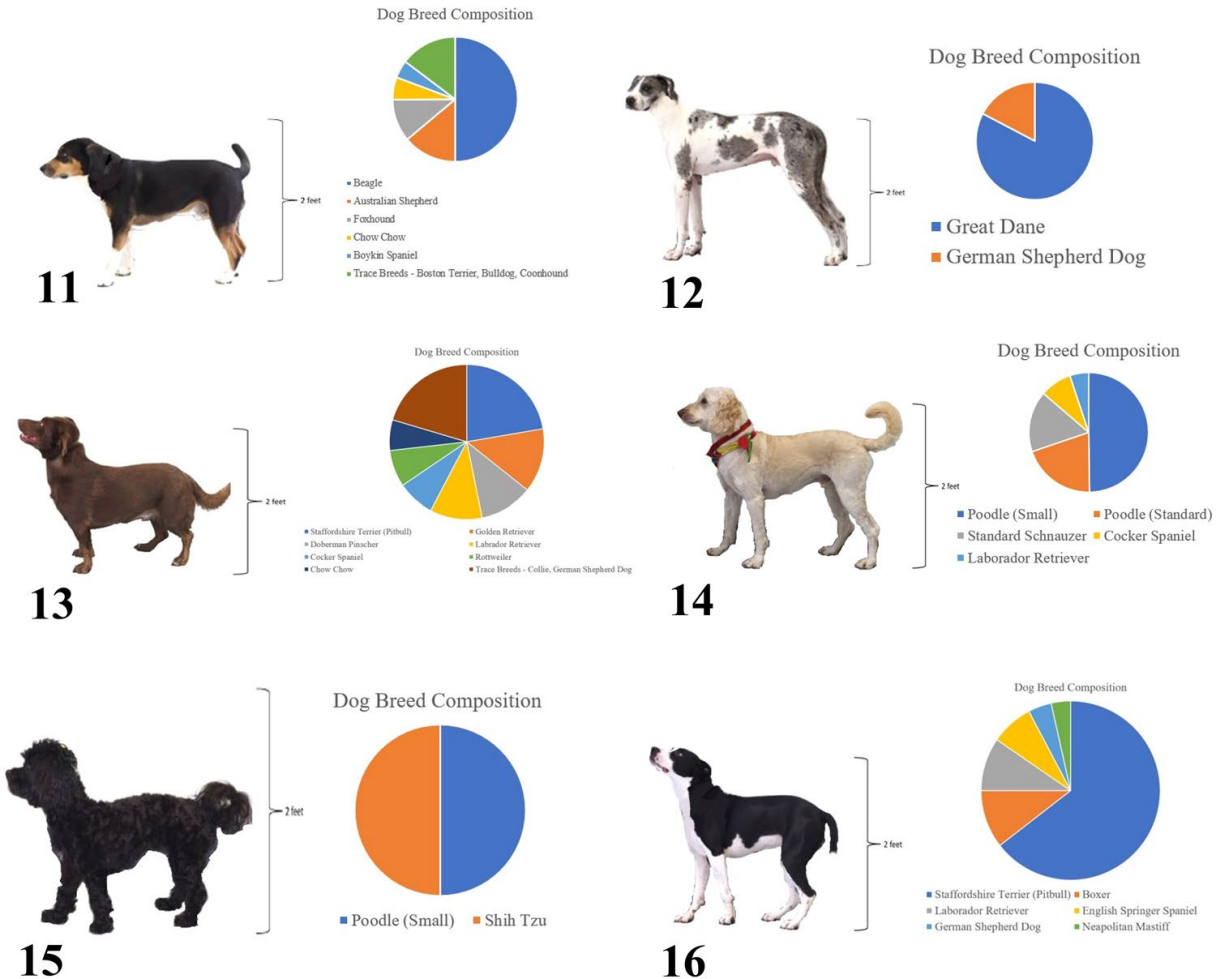
Standardized pictures of six mixed breed dogs with Embark DNA profiles. Presentation of DNA profiles was randomly assigned to survey participants. Form A presented dogs 11, 12, and 13 with their DNA profile and presented dogs 14, 15, and16 without their DNA profile. Form B presented dogs 11, 12, and 13 without their DNA profile and presented dogs 14, 15, and 16 with their DNA profile.

In order to evaluate the effect of breed makeup on these ratings, two forms of the survey were created (A and B). In form A, respondents saw a pie chart representing the breed composition (results of an Embark® DNA panel) for the first three mixed-breed dogs (11–13) while seeing only pictures of the second three mixed-breed dogs (14–16); form B showed the breed composition for the second three mixed-breed dogs (14–16) and pictures for the first three (11–13) (Fig. 2).

The fifth survey block asked respondents to rate how warm or cool they felt toward a list of breeds/groups that included the ten breeds included in the survey and four groups (small/toy dogs, medium/large dogs, purebred dogs, mixed-breed dogs). Respondents indicated their responses on this “Feelings thermometer” by moving a slider along a scale from 0 to 100 and were instructed that, “Ratings between 0 and 49 mean that you do not feel particularly warm or favorable toward the group. A rating of 50 means you feel neutral, neither warm nor cold toward the group. Ratings between 51 and 100 mean that you feel warm or favorable toward the group.” Finally, the sixth block asked respondents to complete demographic questions and the seventh had space for respondents to provide any general feedback. Demographic questions were different for the different respondent populations, with mTurk respondents asked to provide their age, race/ethnicity, gender, region of residence in the United States, highest level of education, and annual household income; academic populations were asked to provide their year in school, major (undergraduates only), whether they were planning to pursue a veterinary degree (undergraduates only), if they had previous experience working at a veterinary clinic (undergraduates and veterinary students), specialty (if applicable), and degrees obtained. Faculty/staff with veterinary degrees were also asked how long it had been since they graduated and where (regionally) they had obtained their veterinary degrees.

### Statistical analysis

Data analysis included descriptive and inferential statistics. Descriptive statistics were calculated for all demographic questions and examined by participant population. Participant populations included general public, undergraduates, 1st and 2nd-year veterinary students (1st/2nd), 3rd and 4th-year veterinary students (3rd/4th), and veterinary faculty and staff. The decision to group 1st and 2nd-year veterinary students and 3rd and 4th-year veterinary students was made in order to best classify veterinary students based on their course work. Across veterinary schools, the 1st and 2nd-year curriculum is focused largely on didactic material while the 3rd and 4th years engage students in applied or clinical learning and may represent a change in views that is important to capture^31^.

Linear mixed effects regression models (R software, R Core Team) were used to assess survey form and its interaction with population and breed on likelihood of adoption and trust ratings (from 0 – 10) accounting for a random effect for subject and university. An ANOVA using Satterthwaite’s approximation to calculate degrees of freedom was used to evaluate all of the adoption/trust ratings together to assess the effect of population, breed, and their interaction. This method was then used to further evaluate the effect of feelings thermometer scores, participant population, and their interaction for each of the likelihood of adoption and trust ratings. Likelihood ratio tests comparing nested linear mixed effects models were used to compare models. Linear mixed models were used to assess likelihood of adoption and trust ratings as predicted by the interaction between the feelings thermometer scores and participant population; in these models, subject and university were accounted for as random effects. When making pairwise comparisons among populations or breeds, linear contrasts were used. Tests of individual contrasts used z-tests, while global tests used Wald tests.

## Results

### Demographics

The final sample included in the analysis was 1020 general public members, 361 undergraduates, 308 1st/2nd year veterinary students, 228 3rd/4th year veterinary students, and 298 veterinary faculty/staff members. Responses were collected from eight veterinary schools; however, insufficient responses were gathered from one school (< 10 responses per group) so data from seven schools were included in the analyses. Responses by respondent category are shown in Supplementary Table S1.

General public respondents were diverse with respect to age, gender, race/ethnicity, and income, but had higher than expected levels of education (see Supplementary Table S2). Undergraduates had a higher proportion of females, but are reflective of the majors included in our sampling procedures^24–26^. Approximately 50% of undergraduate respondents indicated that they were considering pursuing a veterinary medical degree, and 45.5% reported prior veterinary clinical experience. Veterinary academic populations largely reflected the demographics of the profession. Full details on demographics can be found in the supplementary materials (Supplementary Tables S3–S5).

### Seeing DNA profiles affects trust ratings for certain mixed-breed dogs

The survey form respondents received (Form A versus Form B) was associated with trust ratings for likelihood of adoption (χ^2^ (6) = 30.665, *p* < 0.001), trust with a cat (χ^2^ (6) = 16.634, *p* = 0.0107), trust in a crowd (χ^2^ (6) = 13.616, *p* = 0.0342), and trust in a park (χ^2^ (6) = 40.003, *p* < 0.001). When breed composition was displayed for mixed breed dogs, dogs numbered 14, 15, and 16 were rated lower for some of the trust variables; these differences and breed descriptions are shown in Table 1. Contrary to our prediction, there were no form by population interactions (*p* > 0.05).

**Table 1:** Results of linear mixed effects models comparing trust ratings by form for mixed breed dogs. Comparisons were made relative to Form A (negative values mean lower trust in Form B). In Form A, DNA profiles were shown for dogs 11, 12, and 13; in Form B, DNA profiles were shown for dogs 14, 15, and 16. Differences of *p* < 0.05 are highlighted in bold.

Dog number and top two breeds in DNA analysis		Likelihood of adoption	Trust with children	Trust with a cat	Trust in a crowd	Trust in a park
Dog 11 (Beagle, Australian shepherd)	Contrast	−0.023	−0.079	−0.078	−0.072	−0.069
	95% CI	(−0.262,0.215)	(−0.282,0.124)	(−0.282,0.125)	(−0.260,0.117)	(−0.262,0.124)
Dog 12 (Great Dane, German shepherd)	Contrast	−0.152	−0.023	0.046	0.017	0.163
	95% CI	(−0.390,0.086)	(−0.226,0.180)	(−0.157,0.249)	(−0.172,0.206)	(−0.030,0.356)
Dog 13 (no majority breed)	Contrast	−0.026	−0.027	−0.027	0.028	0.055
	95% CI	(−0.264,0.212)	(−0.230,0.176)	(−0.231,0.176)	(−0.161,0.217)	(−0.138,0.249)
Dog 14 (Poodle, schnauzer, cocker spaniel)	Contrast	−**0.262**	−0.136	−0.110	−**0.200**	−0.108
	95% CI	(−0.501,−0.024)	(−0.339,0.067)	(−0.313,0.094)	(−0.388,−0.011)	(−0.301,0.085)
Dog 15 (Poodle, shih tzu)	Contrast	−**0.505**	−**0.270**	−0.120	−0.178	−**0.337**
	95% CI	(−0.744,−0.267)	(−0.473,−0.067)	(−0.323,0.084)	(−0.367,0.010)	(−0.530,−0.144)
Dog 16 (Pitbull, Boxer, GSD, others)	Contrast	0.021	−.082	−**0.296**	−0.111	−0.061
	95% CI	(−0.217,0.260)	(−0.285,0.121)	(−0.499,−0.092)	(−0.299,0.078)	(−0.254,0.132)

### Population-level differences were found in feelings thermometers and trust ratings

As described in the companion article (Caddiell et al.), there was an effect of population on feelings thermometer (FT) ratings, F (3, 22,151) = 28.750,* p* < 0.001. There was no difference between undergraduates and general public respondents (*p* > 0.05), but veterinary students and veterinary faculty and staff reported lower FT ratings across dog breeds compared to the general public (*p* < 0.001). Within academic populations, undergraduate students reported warmer ratings than the veterinary populations (*p* < 0.001) and 1st and 2nd year students reported warmer ratings than the veterinary faculty and staff (*p* = 0.002). No differences were found between FT ratings for the 3rd and 4th year students and the veterinary faculty and staff (*p* > 0.05).

When adoption / trust ratings were modeled together, there was a strong interaction effect among population, dog breed, and the trust ratings (F (180, 139,211) = 9.18, *p* < 0.001) motivating evaluations of each parameter individually by population and breed.

**Figure 3:**
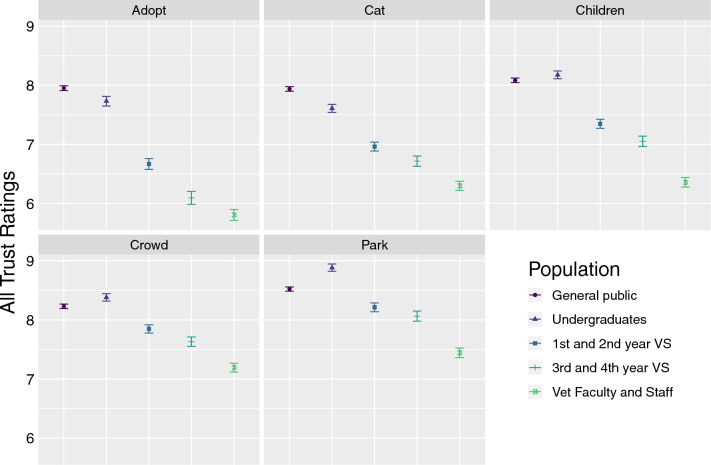
Population means and 95% confidence intervals for likelihood of adoption (‘Adopt’), and trust with cats (‘Cat’), children (‘Children’), crowds (‘Crowd’) and while in park (‘Park’). VS = Veterinary student. There was an effect of population on ratings of likelihood of adoption and trust for each parameter.

There were differences between populations in likelihood of adoption and all trust parameters (Table 2). General public and undergraduate responses were not different from each other with the exception of lower trust ratings by the undergraduates for trust with a cat (t = −2.998, *p* = 0.003) and higher trust ratings by the undergraduates for trust in a park (t = 3.441, *p* < 0.001). Within academic populations, differences were found across adoption and all trust ratings; these are visualized in Fig. 3, with full results in Supplementary Tables S6–S15. Effects of breed are discussed in a later section.

**Table 2:** Results from linear mixed effects models for likelihood of adoption and trust ratings across populations. Significant differences were found for adoption and all trust parameters (differences of *p* < 0.05 are highlighted in bold).

	Wald Test ${\chi }^{2}$ (DF = 3)	*p*-value
Trust parameter		
Likelihood of adoption	165.6	**1.14e**−**35**
Trust with children	163.9	**2.68e**−**35**
Trust with cats	76.45	**1.78e**−**16**
Trust in a crowd	77.38	**1.12e**−**16**
Trust in a park	98.72	**2.93e**−**21**

### Feelings thermometer ratings predict likelihood of adoption and trust ratings for each population

Across all populations, FT ratings predict likelihood of adoption and trust ratings with higher FT ratings predicting higher ratings for adoption and trust parameters (Table 3). For each parameter, there was an interaction among population and FT (Table 3).

**Table 3:** Results of ANOVA models for the effects of population, feelings thermometer ratings, and the interaction among population and feelings thermometer ratings on adoption and trust parameters. Full model included population, feelings thermometer, breed, and each interaction; model results discussed separately. Differences of *p* < 0.05 are highlighted in bold.

		Parameter				
		Likelihood of adoption	Trust with children	Trust with cat	Trust in a crowd	Trust in a park
Population	F-statistic	104.915	16.312	10.791	7.637	12.764
	*p*-value	**2.623e**−**7**	**0.0007**	**0.004**	**0.012**	**0.005**
Feelings thermometer	F-statistic	11,529.840	3239.586	2006.13	2839.843	2372.788
	*p*-value	** < 2.2e**−**16**	** < 2.2e**−**16**	** < 2.2e**−**16**	** < 2.2e**−**16**	** < 2.2e**−**16**
Population: Feelings thermometer	F-statistic	214.588	4.249	4.374	4.357	3.311
	*p*-value	** < 2.2e**−**16**	**0.002**	**0.002**	**0.002**	**0.010**

**Figure 4:**
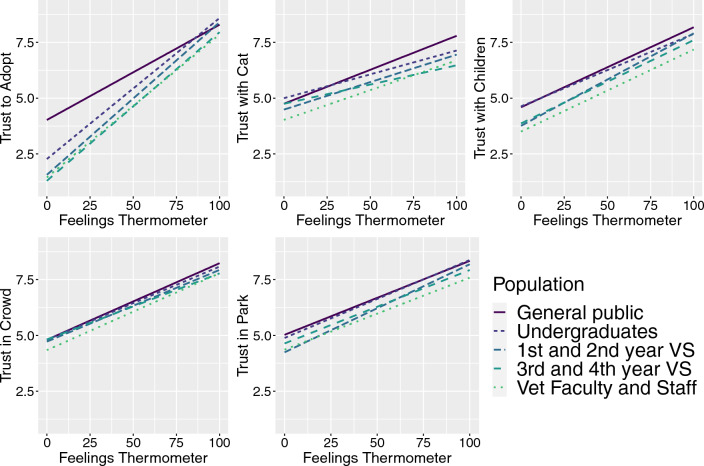
Relationship among feelings thermometer ratings and likelihood to adopt and trust parameters by population. Higher feelings thermometer ratings (x-axis) predict higher ratings of trust with children for all populations.

While all interactions were positive, with higher FT ratings predicting higher adoption/trust ratings, the strength of the interaction differed by population for each parameter (visualized in Fig. 4). The general public was different from all academic populations for all comparisons, with the exception of undergraduates for “trust with a cat”. Within the academic populations, significant population differences were found for likelihood to adopt and all trust ratings (Fig. 4; Supplementary Tables S16–S25). Among the academic populations, the interaction between FT and trust ratings is different overall for likelihood of adoption (F = 11.28, *p* < 0.001), trust with cats (F = 9.33, p < 0.001), trust with children (F = 10.35, < 0.001), and trust in a park (F = 5.66, < 0.001), but not for trust in a crowd (F = 2.55, *p* = 0.054).

### Populations differ in their likelihood of adoption and trust ratings by dog breed

**Figure 5:**
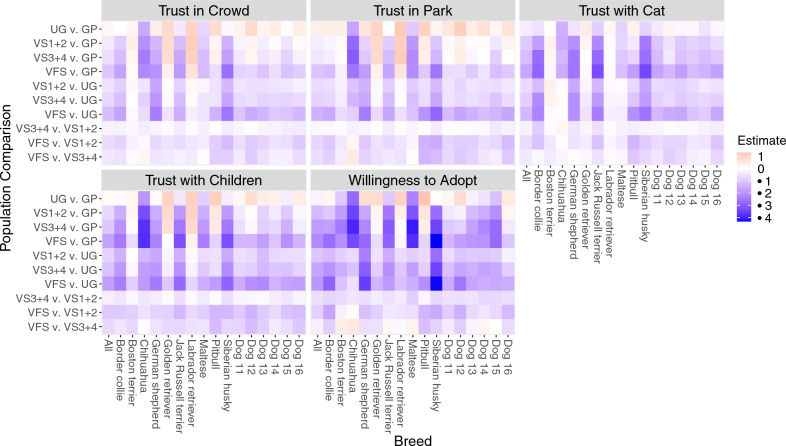
Heat map visualization of the estimated average difference in ratings for each adoption/trust parameter between the first listed population compared to the second listed population; red tones indicate that the first population rated the breed higher in trust than the second population on that parameter, while blue tones indicate that the first population rated the breed lower in trust than the second population. The following participant population abbreviations were used: GP = general public, UG = undergraduates; VS1 + 2 = 1st and 2nd year veterinary students, VS3 + 4 = 3rd and 4th year veterinary students, and VFS = veterinary faculty and staff. Dog 11 = Beagle, Australian shepherd; Dog 12 = Great Dane, German shepherd; Dog 13 = Staffordshire terrier (pitbull), trace breeds; Dog 14 = Poodle (small), poodle (standard); Dog 15 = Poodle (small), shih tzu; Dog 16 = Staffordshire terrier (pitbull), Boxer.

Across all populations, there was an effect of breed on likelihood of adoption and trust ratings with significant breed by population interactions for all parameters (Table 4). Model results for population differences for each breed are available as Supplementary CSV data files. Differences can be visualized in Fig. 5.

**Table 4:** Results of ANOVA models for the effects of population, breed, and the interaction among population and breed ratings on adoption and trust parameters. Full model included population, feelings thermometer, breed, and each interaction; model results discussed separately. Differences of *p* < 0.05 are highlighted in bold.

		**Parameter**				
		Likelihood of adoption	Trust with children	Trust with cat	Trust in a crowd	Trust in a park
Breed	F-statistic	10.606	69.902	105.908	72.158	70.090
	*p*-value	** < 2.2e**−**16**	** < 2.2e**−**16**	** < 2.2e**−**16**	** < 2.2e**−**16**	** < 2.2e**−**16**
Population: Breed	F-statistic	4.142	9.230	7.498	6.260	7.615
	*p*-value	**1.212e**−**15**	** < 2.2e**−**16**	** < 2.2e**−**16**	** < 2.2e**−**16**	** < 2.2e**−**16**

### Clinical experience affects likelihood of adoption and trust ratings among undergraduates

**Figure 6:**
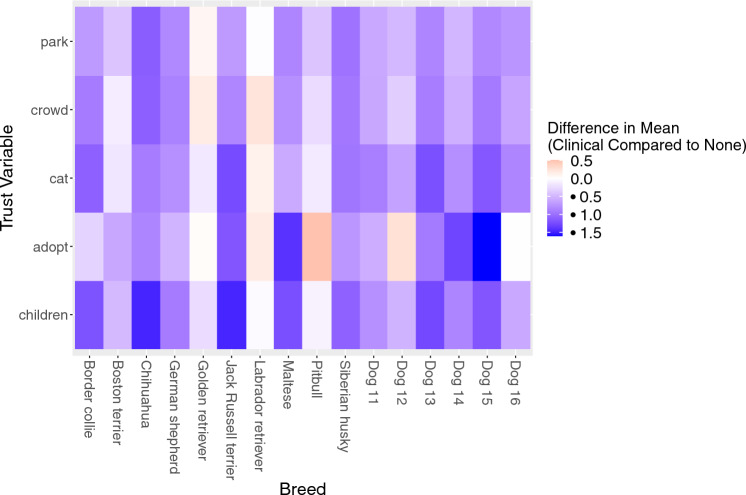
Heat map displaying the differences in mean trust ratings between undergraduates with and without veterinary clinical experience for each dog breed. Heat map visualization of the estimated average difference in ratings for each adoption/trust parameter between undergraduates with and without clinical experience. Red tones indicate respondents with veterinary clinical experience rated the breed higher than respondents without veterinary clinical experience on that parameter, while blue tones indicate that respondents with clinical veterinary experience rated the breed lower than respondents without veterinary clinical experience on that parameter. Dog 11 = Beagle, Australian shepherd; Dog 12 = Great Dane, German shepherd; Dog 13 = Staffordshire terrier (pitbull), trace breeds; Dog 14 = Poodle (small), poodle (standard); Dog 15 = Poodle (small), shih tzu; Dog 16 = Staffordshire terrier (pitbull), Boxer.

Among the undergraduate population, there was an effect of veterinary clinical experience on certain trust ratings, with interactions among veterinary clinical experience and breed for all comparisons (Table 5). Differences in mean ratings for trust parameters are visualized in Fig. 6, with full results available in Supplementary Tables S26–S30.

**Table 5:** Results of linear mixed effects models for undergraduates with and without clinical veterinary experience. Results are shown for each trust parameter. Differences of *p* < 0.05 are highlighted in bold.

		Parameter				
		Likelihood of adoption	Trust with children	Trust with cat	Trust in a crowd	Trust in a park
Clinical experience	F-statistic	7.784	24.396	14.781	12.074	14.345
	*p*-value	**0.006**	**1.204 e**−**6**	**0.0001**	**0.0006**	**0.0002**
Breed	F-statistic	128.719	89.934	41.052	77.224	103.493
	*p*-value	** < 2.2e**−**16**	** < 2.2e**−**16**	** < 2.2e**−**16**	** < 2.2e**−**16**	** < 2.2e**−**16**
Clinical experience:Breed	F-statistic	6.940	6.060	4.653	5.221	3.468
	*p*-value	**3.183e**−**15**	**8.944e**−**13**	**5.629e**−**9**	**1.728e**−**10**	**6.026e**−**6**

## Discussion

These results support our predictions that there are differences in feelings thermometer (FT), likelihood of adoption, and trust ratings among members of the general public, undergraduates, and academic veterinary populations. Further, many of these ratings change in a direction supporting our prediction that increased exposure to veterinary training and clinical experience are associated with ratings that more closely resemble those of veterinarians compared to members of the general public. These findings are important in understanding the relationship between veterinary training and beliefs about dogs, while suggesting a directional effect on development of these beliefs. While ratings for FT, likelihood of adoption, and trust were overall lower in the veterinary academic populations than in the general public or undergraduate respondents, there were strong effects of breed on these differences. We also found differences between undergraduates with and without clinical veterinary experience, supporting our premise that veterinary training affects beliefs about dog breeds. While many previous studies have found that stereotypes about behavior exist regarding dog breeds, this is the first to evaluate differences among these important populations, and to investigate how feelings of warmth affect ratings of trust.

For mixed breed dogs, we predicted that seeing the photos with DNA profiles (versus the photos alone) would affect ratings of trust and likelihood of adoption, with ratings shifted in the same direction (more or less trust) as the predominant breed when that breed (or type) was also represented in the survey. However, these predictions were not fully supported, with differences found for only some of the trust ratings, and no differences among populations. Marked differences were found when respondents saw the breed composition for the dog with 50% small poodle and 50% shih tzu; seeing the DNA profile was associated with lower likelihood of adoption, trust with children, and trust in a park. Seeing the DNA profile was also associated with lower trust in a crowd for the dog with contributions from poodle (standard and small), schnauzer, cocker spaniel, and Labrador retriever. Results for these were not expected and require additional future evaluation. Based on previous work, the strongest response was expected for the dog whose breed composition had a majority of pitbull type dog as this label has been shown to affect ratings of behavioral traits in dogs and lengthen shelter stays^6^. In our study, two dogs had pitbull as part of their breed composition; one was highly mixed with no majority breed, while the other was over 50% pitbull-type breed. The dog with the highest percentage of pitbull DNA was rated as significantly less trusted with a cat when respondents saw the breed composition compared to those who didn’t, however no other comparisons for these two dogs were significant. In a large study, Morill found that breed-based biases were less frequently applied when respondents viewed a mixed-breed dog^7^, and this may have affected our findings. However, Clarke et al. found that when a mixed breed dog was labeled as a “terrier” vs. “toy” type dog, this affected ratings for playfulness, curiosity and fearlessness, propensity to chase, sociability, and aggressiveness^15^. In that study, an ambiguous dog breed (the “Tiskita”) was given one of two labels, rather than being displayed with a breed composition (vs. none) as done in our study. In our study, respondents likely guessed at breed when making their ratings, which has been demonstrated to be frequently inaccurate, even among people who regularly work with dogs^7,15,27^ but may be more accurate when dogs are from a common breed or have a high percentage of pitbull-type DNA^7^. In future work, selection of pictures of dogs where breeds were also depicted in the rest of the survey would allow more direct comparison with the population ratings for the trust variable.

For three of the trust ratings, undergraduates and general public respondents were not different from one another; the exceptions were trust with a cat (where undergraduates had significantly lower trust ratings than the general public) and trust in a park, where undergraduates had significantly higher trust ratings than the general public. While average ratings across all breeds were similar between the two populations, there were significant effects of breed, shown in the heat maps, that reveal important differences in the ratings. Across all trust variables, undergraduate respondents rated pitbulls higher (more trustworthy) than the general public respondents, with the highest estimate for likelihood of adoption. German shepherds were also rated higher by undergraduates than general public respondents for trust in a park and likelihood of adoption. The opposite was true for Chihuahuas and Malteses, where undergraduate ratings of trust were lower than the general public ratings for all trust variables and likelihood of adoption. While undergraduate respondents also had higher ratings than the general public respondents for trust parameters and likelihood of adoption of golden and Labrador retrievers, the other breed differences are intriguing as they are opposite to the common perception, particularly for pitbulls. The population of undergraduates surveyed likely played a role in these findings, as the sampling targeted majors with an interest in animal health; nearly half of the undergraduate respondents had veterinary clinical experience, further affecting ratings as discussed below.

When comparing the veterinary academic population to the other populations, the findings of overall lower—regardless of breed—FT, likelihood of adoption, and trust ratings among the veterinary academic populations (compared to the general public and undergraduate respondents) may be logical given that veterinary training includes exposure to many more dogs than experienced by most members of the general public, and in situations in which dogs are more likely to show fearful or anxious behaviors^28,29^. Dogs may also be presented to the clinic/hospital following injuries or traumatic events, which can shape veterinarian views on their behavior based both on their response to the situation and the setting of their injury (i.e. if the injury was caused by a fight with another dog). However, this remains an area for further exploration as both human and veterinary medicine studies have shown that clinical exposure is associated with lower empathy^30,31^, and previous work found that veterinary feelings of warmth toward a dog breed were inversely related to their ratings of pain sensitivity^16^. Here, we found strong, positive relationships between FT ratings and ratings of trust. Intuitively, this makes sense as warmer feelings would be expected toward dogs that an individual trusted in a variety of situations. Observation of the slope of this relationship (Fig. 4) reveals differences among populations, particularly for likelihood of adoption and trust with children which are markedly different for the academic populations, particularly at the lower (cooler) end of the feelings thermometer ratings. Importantly for our predictions regarding veterinary training, significant differences were found *within* the veterinary academic populations for the FT and most trust variables. While not all were different among the veterinary participant populations, the ordering of the responses was always in the predicted direction (with higher trust ratings from 1st/2nd years, lower from 3rd/4th year students, and the lowest from veterinary faculty/staff). Further, veterinary clinical experience among undergraduates was associated with overall lower ratings of trust similar to what was found among the veterinary academic populations. Of particular interest are ratings for trust with children and likelihood of adoption, where the largest differences among populations (and largest effects of veterinary clinical experience) were seen. Further work is needed to fully understand the reasons for these differences; however, these may be variables of particular salience in the veterinary academic population. While our survey did not ask about the presence of children in the home, previous work has shown that being a parent affects some ratings of dog behaviors among veterinarians^17^, particularly highlighting gender differences. For example, Kogan et al. found differences in agreement with the statement that “some dog breeds should be banned from being around children”; among respondents who did not own a pit bull, 26.9% of men with children and 19.7% of men without children agreed with this statement compared to 12.6% of females with children and 7.4% of females without children^17^.

It is impossible to determine “truth” regarding trustworthiness of a given dog breed, particularly as research strongly supports within-breed variability that exceeds among-breed variability^7,11^. However, some data are available to support or refute certain perceptions. In a study by Duffy et al. (2008), nearly 4,000 owners of dogs were surveyed using the Canine Behavioral Research Questionnaire (C-BARQ), and 33 breeds (or breed types) with at least 45 responses each were compared for stranger-directed aggression, owner-directed aggression, dog-directed aggression (toward unfamiliar dogs), and dog-rivalry (defined as dog-directed aggression toward another household dog). Using scores averaged across all dogs, they found that Chihuahuas scored higher in all forms of aggression, while golden and Labrador retrievers were generally below average (both were approximately average for owner-directed aggression). Pitbulls were rated as below average for owner-directed aggression, average for stranger-directed aggression, and above average for dog-directed aggression and dog rivalry^5^. This dataset and behavioral phenotypes were included in genetic studies that identified genetic loci associated with fear and aggression^13^. These authors found that certain types of fearful and aggressive behaviors cluster together (fear and/or aggression toward strangers and unfamiliar dogs) and identified genetic loci associated with certain fear/aggressive behavioral phenotypes. They noted an association between several fear/aggression behavioral phenotypes and small body size^13^; this finding was supported in further work by the same group^32^. Their later study also found that pitbull-type dogs were no more likely to have a diagnosed behavioral condition than any other included breed^32^. Maclean et al. (2019) also used C-BARQ and genetic data to demonstrate high heritability of behavioral traits between breeds. While ranking of breeds across behavioral domains was not part of their study, they found higher heritability than had previously been reported^14^. Our study asked about perceptions of trust, which may reflect a person’s belief about a dog’s potential behavior in a given setting, but are not equivalent to the behavioral phenotypes included in the C-BARQ. However, it is interesting to note similarities between the owner ratings of behaviors via the C-BARQ and the similarities with ratings of trust from the veterinary academic populations.

Several limitations of our study should be discussed. First, our veterinary sample had an overrepresentation of females, and may not fully represent the views of male veterinarians or veterinary students. Veterinary faculty, staff, residents, and interns were grouped together as veterinary faculty/staff; this both increased power and was justified as they work together in the teaching hospital and are integral to the clinical education of students. However, this precludes evaluation of the role of faculty or specialty, and future work will be needed to evaluate this with greater granularity. The cross-sectional nature of this study also precludes us from determining exactly how, or when, changes in beliefs occur. This study established that there are differences between populations, even within veterinary academic populations, and future longitudinal work will be needed.

## Conclusions

In summary, we found that ratings of warmth, trust, and likelihood of adoption of dogs were different among the surveyed populations, and that veterinary training and clinical exposure were associated with differences in ratings. Knowledge of breed composition for mixed-breed dogs affected some ratings of trust, but did not vary by population. Veterinary training and clinical exposure were associated with lower ratings overall for most breeds, with notable exceptions for golden and Labrador retrievers and pitbulls. We have demonstrated that feelings of warmth and ratings of trust are different between veterinarians and general public members and found evidence supporting cultural transmission of these beliefs during veterinary training and clinical exposure. Future work is needed to understand whether these beliefs impact care of dogs, and on how these beliefs are shaped over time.

### Supplementary Information

Supplementary Information 1.

Supplementary Information 2.

Supplementary Information 3.

Supplementary Information 4.

Supplementary Information 5.

Supplementary Information 6.

## Data Availability

The datasets are available from the corresponding author on reasonable request; many are included as supplementary files to this manuscript.
