# Supplementary material for: Veterinary education and experience shape beliefs about dog breeds. Part 2: Trust
Source: Sci Rep. 2023 Aug 24;13:13847. doi: 10.1038/s41598-023-40464-3 (PMC10449930; doi:10.1038/s41598-023-40464-3)
Supplement: Supplementary file 6 — Supplementary Information 6. [file 41598_2023_40464_MOESM6_ESM.docx]

**Supplementary Table S1.** Demographic characteristics (gender, race and/or ethnicity, age) of the sample by population. The following abbreviations were used: VS = student and vet = veterinary.

|  | Participant Population  No. (%) | | | | |
| --- | --- | --- | --- | --- | --- |
| Characteristic | General public | Undergraduates | 1^st^ and 2^nd^ Year VS | 3^rd^ and 4^th^ Year VS | Vet Faculty and Staff |
| *Gender* | | | | | |
| Female | 463 (45.4%) | 302 (83.7%) | 178 (91.8%) | 112 (83.6%) | 239 (81.6%) |
| Male | 556 (54.5%) | 54 (15.0%) | 14 (7.2%) | 22 (16.4%) | 49 (16.7%) |
| Other | 1 (0.1%) | 4 (1.1%) | 1 (0.5%) | 0 (0%) | 2 (0.7%) |
| Prefer not to say | 0 (0%) | 0 (0%) | 1 (0.5%) | 0 (0%) | 3 (1.0%) |
| *Race / Ethnicity* | | | | | |
| Asian | 62 (6.1%) | 16 (4.4%) | 9 (4.6%) | 6 (4.5%) | 5 (1.7%) |
| Black or African American | 101 (9.9%) | 23 (6.4%) | 5 (2.6%) | 3 (2.2%) | 8 (2.7%) |
| Caucasian | 745 (73.0%) | 272 (75.3%) | 154 (79.4%) | 112 (83.6%) | 256 (87.4%) |
| Hispanic, Latino or Spanish origin | 60 (5.9%) | 12 (3.3%) | 15 (7.7%) | 7 (5.2%) | 9 (3.0%) |
| Native American or American Indian | 42 (4.1%) | 1 (0.3%) | 2 (1.0%) | 1 (0.7%) | 1 (0.3%) |
| Pacific Islander | 0 (0%) | 0 (0%) | 0 (0%) | 0 (0%) | 1 (0.3%) |
| From multiple races | 10 (1.0%) | 32 (8.9%) | 8 (4.1%) | 5 (3.7%) | 7 (2.4%) |
| Prefer not to say | 0 (0.0%) | 1 (0.3%) | 1 (0.5%) | 0 (0%) | 6 (2.0%) |
| *Age* | | | | | |
| 18 – 29 years | 225 (22.1%) | 357 (98.9%) | 174 (89.7%) | 122 (91.0%) | 54 (18.4%) |
| 30 – 39 years | 368 (36.1%) | 3 (0.8%) | 18 (9.3%) | 11 (8.2%) | 73 (24.9%) |
| 40 – 49 years | 232 (22.7%) | 0 (0%) | 1 (0.5%) | 1 (0.7%) | 48 (16.4%) |
| 50 – 59 years | 126 (12.4%) | 0 (0%) | 0 (0%) | 0 (0%) | 62 (21.2%) |
| 60 – 69 years | 60 (5.9%) | 0 (0%) | 0 (0%) | 0 (0%) | 35 (11.9%) |
| 70 or older | 9 (0.9%) | 0 (0%) | 0 (0%) | 0 (0%) | 10 (3.4%) |
| Prefer not to say | 0 (0%) | 1 (0.3%) | 1 (0.5%) | 0 (0%) | 11 (3.7%) |

**Supplementary Table S2.** Additional demographic characteristics (geographic region, annual household income, highest level of education) of the general population sample.

| Characteristic | No. (%) |
| --- | --- |
| *Geographic Region* | |
| Midwest | 191 (18.7%) |
| Northeast | 220 (21.6%) |
| South | 360 (35.3%) |
| West | 249 (24.4%) |
| *Annual Household Income* | |
| ≤ $24,999 | 84 (8.2%) |
| $25,000 - $39,999 | 147 (14.4%) |
| $40,000 - $59,999 | 276 (27.1%) |
| $60,000 - $79,999 | 223 (21.9%) |
| $80,000 - $99,999 | 149 (14.6%) |
| $100,000 - $149,999 | 103 (10.1%) |
| $150,000 - $199,999 | 26 (2.5%) |
| ≥ $200,000 | 12 (1.2%) |
| *Highest Level of Education* |  |
| Some high school, no diploma | 3 (0.3%) |
| High school graduate, diploma, or equivalent | 65 (6.4%) |
| Some college credit, no degree | 97 (9.5%) |
| Trade / technical / vocational training | 24 (2.4%) |
| Associate degree | 82 (8.0%) |
| Bachelor’s degree | 566 (55.5%) |
| Master’s degree | 176 (17.3%) |
| Doctorate or professional degree | 7 (0.7%) |

**Supplementary Table S3.** Characteristics of the undergraduate sample.

| Characteristic | No. (%) |
| --- | --- |
| *Major* | |
| Animal Sciences | 181 (50.1%) |
| Biology | 109 (30.2%) |
| Zoology | 45 (12.5%) |
| Other | 26 (7.2%) |
| *Year in undergraduate degree program* | |
| 1^st^ Year | 47 (13.0%) |
| 2^nd^ Year | 78 (21.6%) |
| 3^rd^ Year | 117 (32.4%) |
| 4^th^ Year | 109 (30.2%) |
| 5^th^ Year | 9 (2.5%) |
| 6^th^ Year or More | 1 (0.3%) |
| *Considering pursuing a veterinary degree post-graduation* |  |
| Yes | 187 (51.8%) |
| No | 174 (48.2%) |
| *Previous experience working in a veterinary clinic* |  |
| Yes | 164 (45.4%) |
| No | 197 (54.6%) |

**Supplementary Table S4.** Characteristics of the veterinary student sample. The abbreviation VS = veterinary student was used.

|  | Veterinary Student Participant Population  No. (%) | |
| --- | --- | --- |
| Characteristic | 1^st^ and 2^nd^ Year VS | 3^rd^ and 4^th^ Year VS |
| *University attending for veterinary school* | | |
| Auburn | 24 (7.8%) | 13 (5.7%) |
| LSU | 32 (10.4%) | 15 (6.6%) |
| NCSU | 88 (28.6%) | 98 (43.0%) |
| Oregon | 43 (14.0%) | 18 (7.9%) |
| Tufts | 37 (12.0%) | 26 (11.4%) |
| UGA | 44 (14.3%) | 21 (9.2%) |
| V-Tech | 40 (13.0%) | 37 (16.2%) |
| *Year in veterinary school* | | |
| 1^st^ Year | 164 (53.2%) | - |
| 2^nd^ Year | 144 (46.8%) | - |
| 3^rd^ Year | - | 118 (51.8%) |
| 4^th^ Year | - | 110 (48.2%) |
| *Areas of veterinary practice interest* | | |
| Small animal | 191 (62.0%) | 149 (65.4%) |
| Mixed animal | 137 (44.5%) | 62 (27.2%) |
| Food animal | 41 (13.3%) | 36 (15.8%) |
| Zoo / Wildlife | 76 (24.7%) | 45 (19.7%) |
| Laboratory animal | 17 (5.5%) | 18 (7.9%) |
| Undecided | 26 (8.4%) | 4 (1.8%) |
| *Previous experience working in a veterinary clinic* | | |
| Yes | 284 (92.2%) | 214 (93.9%) |
| No | 24 (7.8%) | 14 (6.1%) |

**Supplementary Table S5.** Characteristics of the veterinary faculty and staff sample.

| Characteristic | No. (%) |
| --- | --- |
| *University employed by* |  |
| Auburn | 34 (11.6%) |
| LSU | 16 (5.5%) |
| NCSU | 173 (59.0%) |
| Oregon | 16 (5.5%) |
| Tufts | 53 (18.1%) |
| UGA | 1 (0.3%) |
| V-Tech | 0 (0.0%) |
| *Veterinary specialty / area of expertise employed in* |  |
| Administration | 14 (4.8%) |
| Anatomy | 3 (1.0%) |
| Anaesthesia | 16 (5.5%) |
| Animal welfare and behavior | 7 (2.4%) |
| Dermatology | 3 (1.0%) |
| Emergency and critical care | 27 (9.2%) |
| Exotics / wildlife | 5 (1.7%) |
| Food animal / large animal medicine | 9 (3.1%) |
| Hospitalized patient care | 4 (1.4%) |
| Infectious disease | 3 (1.0%) |
| Internal medicine including cardiology, neurology, and oncology | 55 (18.8%) |
| Laboratory animal medicine | 19 (6.5%) |
| Microbiology | 6 (2.0%) |
| Nutrition | 3 (1.0%) |
| Ophthalmology | 4 (1.4%) |
| Pathology | 15 (5.1%) |
| Pharmacology | 3 (1.0%) |
| Preventative medicine | 15 (5.1%) |
| Public health | 4 (1.4%) |
| Radiology | 13 (4.4%) |
| Research | 14 (4.8%) |
| Sports medicine and rehabilitation | 4 (1.4%) |
| Surgery | 15 (5.1%) |
| Theriogenology | 10 (3.4%) |
| Other | 22 (7.5%) |
| *Degrees obtained* |  |
| Associate’s degree | 64 (21.8%) |
| Bachelor’s degree | 186 (63.5%) |
| Master’s degree | 72 (24.6%) |
| Doctor of Philosophy (PhD) | 47 (16.0%) |
| Doctor of Veterinary Medicine (DVM) | 146 (49.8%) |
| *For those who have obtained a DVM, how much time has passed since veterinary school graduation* | |
| Less than 1 year | 12 (8.2%) |
| 1 – 5 years | 33 (22.6%) |
| 6 – 10 years | 16 (11.0%) |
| 11 – 15 years | 16 (11.0%) |
| 16 – 20 years | 10 (6.8%) |
| More than 20 years | 59 (40.4%) |
| *For those who have obtained a DVM, region of the United States veterinary degree was obtained from* | |
| Midwest | 29 (19.9%) |
| Northeast | 26 (17.8%) |
| South | 49 (33.6%) |
| West | 16 (10.9%) |
| Outside of the United States | 26 (17.8%) |

**Supplementary Table S6. Likelihood of adoption – General public comparisons.** Participant population group comparisons between the general public and each academic participant population for likelihood of adoption ratings using a linear mixed effects model with fixed effects for participant population and random effects for the individual. Estimate represents the estimated average difference in rating for the academic participant population of interest compared to the general public. Negative estimates indicate that the academic participant populations rated dogs lower (less likely to adopt) compared to the general public participant population. Positive estimates indicate that the academic participant populations rated dogs higher (more likely to adopt) compared to the general public participant population. The following abbreviations were used: VS = student and vet = veterinary.

| Population Comparison | Estimate | Std. Error | z-statistic | *p*-value |
| --- | --- | --- | --- | --- |
| General public v. Undergraduates | -0.218 | 0.114 | -1.910 | 0.056 |
| General public v. 1^st^ and 2^nd^ Year VS | -1.283 | 0.121 | -10.562 | 2.22e-16 |
| General public v. 3^rd^ and 4^th^ Year VS | -1.857 | 0.137 | -13.568 | 2.22e-16 |
| General public v. Vet Faculty and Staff | -2.146 | 0.123 | -17.467 | 2.22e-16 |

**Supplementary Table S7. Likelihood of adoption - Academic population comparisons.** Academic participant population comparisons for likelihood of adoption ratings using a linear mixed effects model with fixed effects for participant population and random effects for the individual. Estimates were obtained using linear contrasts. Estimate represents the estimated average difference in likelihood of adoption rating for the participant population with more training compared to the participant population with less training. Negative estimates indicate that the participant population with more training rated dogs lower (less likely to adopt) compared to the participant population with less training. Positive estimates indicate that the participant population with more training rated dogs higher (more likely to adopt) compared to the participant population with more training. The following abbreviations were used: VS = student and vet = veterinary.

| Population Comparison | Estimate | Std. Error | z-statistic | *p*-value |
| --- | --- | --- | --- | --- |
| Undergraduates v. 1^st^ and 2^nd^ Year VS | -1.064 | 0.145 | -7.346 | 2.03e-13 |
| Undergraduates v. 3^rd^ and 4^th^ Year VS | -1.638 | 0.158 | -10.369 | 2.22e-16 |
| Undergraduates v. Vet Faculty and Staff | -1.927 | 0.146 | -13.197 | 2.22e-16 |
| 1^st^ and 2^nd^ Year VS v. 3rd and 4th VS | -0.574 | 0.163 | -3.517 | 4.36e-4 |
| Vet Faculty and Staff v. 1^st^ and 2^nd^ Year VS | -0.863 | 0.152 | -5.691 | 1.26e-08 |
| Vet Faculty and Staff v. 3^rd^ and 4^th^ Year VS | -0.289 | 0.164 | -1.761 | 0.078 |

**Supplementary Table S8. Trust with children – General public comparisons** Participant population group comparisons between the general public and each academic participant population for trust with children ratings using a linear mixed effects model with fixed effects for participant population and random effects for the individual. Estimate represents the estimated average difference in rating for the academic participant population of interest compared to the general public. Negative estimates indicate that the academic participant populations rated dogs lower (less trust) compared to the general public participant population. Positive estimates indicate that the academic participant populations rated dogs higher (more trust) compared to the general public participant population.

| Population Comparison | Estimate | Std. Error | z-statistic | *p*-value |
| --- | --- | --- | --- | --- |
| General public v. Undergraduates | 0.091 | 0.105 | 0.868 | 0.385 |
| General public v. 1^st^ and 2^nd^ Year VS | -0.736 | 0.112 | -6.591 | 4.35e-11 |
| General public v. 3^rd^ and 4^th^ Year VS | -1.032 | 0.126 | -8.197 | 2.22e-16 |
| General public v. Vet Faculty and Staff | -1.727 | 0.113 | -15.286 | 2.22e-16 |

**Supplementary Table S9. Trust with children - Academic population comparisons.** Academic participant population comparisons for trust with children ratings using a linear mixed effects model with fixed effects for participant population and random effects for the individual. Estimates were obtained using linear contrasts. Estimate represents the estimated average difference in trust rating for the participant population with more training compared to the participant population with less training. Negative estimates indicate that the participant population with more training rated dogs lower (less trust) compared to the participant population with less training. Positive estimates indicate that the participant population with more training rated dogs higher (more trust) compared to the participant population with more training. The following abbreviations were used: VS = student and vet = veterinary.

| Population Comparison | Estimate | Std. Error | z-statistic | *p*-value |
| --- | --- | --- | --- | --- |
| Undergraduates v. 1^st^ and 2^nd^ Year VS | -0.828 | 0.133 | -6.212 | 5.25e-10 |
| Undergraduates v. 3^rd^ and 4^th^ Year VS | -1.123 | 0.145 | -7.729 | 1.09e-14 |
| Undergraduates v. Vet Faculty and Staff | -1.818 | 0.134 | -13.538 | 2.22e-16 |
| 1^st^ and 2^nd^ Year VS v. 3rd and 4th VS | -0.295 | 0.150 | -1.968 | 0.049 |
| Vet Faculty and Staff v. 1^st^ and 2^nd^ Year VS | -0.991 | 0.139 | -7.104 | 1.22e-12 |
| Vet Faculty and Staff v. 3^rd^ and 4^th^ Year VS | -0.695 | 0.151 | -4.604 | 4.15e-06 |

**Supplementary. Table S10. Trust with cats – General public comparisons.** Participant population group comparisons between the general public and each academic participant population for trust with cats ratings using a linear mixed effects model with fixed effects for participant population and random effects for the individual. Estimate represents the estimated average difference in rating for the academic participant population of interest compared to the general public. Negative estimates indicate that the academic participant populations rated dogs lower (less trust) compared to the general public participant population. Positive estimates indicate that the academic participant populations rated dogs higher (more trust) compared to the general public participant population. The following abbreviations were used: VS = student and vet = veterinary.

| Population Comparison | Estimate | Std. Error | z-statistic | *p*-value |
| --- | --- | --- | --- | --- |
| General public v. Undergraduates | -0.326 | 0.109 | -2.998 | 0.003 |
| General public v. 1^st^ and 2^nd^ Year VS | -0.975 | 0.115 | -8.457 | 2.22e-16 |
| General public v. 3^rd^ and 4^th^ Year VS | -1.220 | 0.130 | -9.389 | 2.22e-16 |
| General public v. Vet Faculty and Staff | -1.638 | 0.117 | -14.043 | 2.22e-16 |

**Supplementary Table S11. Trust with cats – Academic population comparisons.** Academic participant population comparisons for trust with cats ratings using a linear mixed effects model with fixed effects for participant population and random effects for the individual. Estimates were obtained using linear contrasts. Estimate represents the estimated average difference in trust rating for the participant population with more training compared to the participant population with less training. Negative estimates indicate that the participant population with more training rated dogs lower (less trust) compared to the participant population with less training. Positive estimates indicate that the participant population with more training rated dogs higher (more trust) compared to the participant population with more training. The following abbreviations were used: VS = student and vet = veterinary.

| Population Comparison | Estimate | Std. Error | z-statistic | *p*-value |
| --- | --- | --- | --- | --- |
| Undergraduates v. 1^st^ and 2^nd^ Year VS | -0.650 | 0.138 | -4.723 | 2.32e-06 |
| Undergraduates v. 3^rd^ and 4^th^ Year VS | -0.894 | 0.150 | -5.962 | 2.49e-09 |
| Undergraduates v. Vet Faculty and Staff | -1.313 | 0.139 | -9.466 | 2.22e-16 |
| 1^st^ and 2^nd^ Year VS v. 3rd and 4th VS | -0.245 | 0.155 | -1.579 | 0.114 |
| Vet Faculty and Staff v. 1^st^ and 2^nd^ Year VS | -0.663 | 0.144 | -4.603 | 4.16e-06 |
| Vet Faculty and Staff v. 3^rd^ and 4^th^ Year VS | -0.418 | 0.156 | -2.681 | 0.007 |

**Supplementary Table S12. Trust in a crowd – General public comparisons.** Participant population group comparisons between the general public and each academic participant population for trust in a crowd ratings using a linear mixed effects model with fixed effects for participant population and random effects for the individual. Estimate represents the estimated average difference in rating for the academic participant population of interest compared to the general public. Negative estimates indicate that the academic participant populations rated dogs lower (less trust) compared to the general public participant population. Positive estimates indicate that the academic participant populations rated dogs higher (more trust) compared to the general public participant population. The following abbreviations were used: VS = student and vet = veterinary.

| Population Comparison | Estimate | Std. Error | z-statistic | *p*-value |
| --- | --- | --- | --- | --- |
| General public v. Undergraduates | 0.151 | 0.099 | 1.521 | 0.128 |
| General public v. 1^st^ and 2^nd^ Year VS | -0.383 | 0.105 | -3.631 | 2.82e-4 |
| General public v. 3^rd^ and 4^th^ Year VS | -0.600 | 0.119 | -5.047 | 4.49e-07 |
| General public v. Vet Faculty and Staff | -1.036 | 0.107 | -9.713 | 2.22e-16 |

**Supplementary Table S13. Trust in a crowd - Academic population comparisons.** Academic participant population comparisons for trust in a crowd ratings using a linear mixed effects model with fixed effects for participant population and random effects for the individual. Estimates were obtained using linear contrasts. Estimate represents the estimated average difference in trust rating for the participant population with more training compared to the participant population with less training. Negative estimates indicate that the participant population with more training rated dogs lower (less trust) compared to the participant population with less training. Positive estimates indicate that the participant population with more training rated dogs higher (more trust) compared to the participant population with more training. The following abbreviations were used: VS = student and vet = veterinary.

| Population Comparison | Estimate | Std. Error | z-statistic | *p*-value |
| --- | --- | --- | --- | --- |
| Undergraduates v. 1^st^ and 2^nd^ Year VS | -0.534 | 0.126 | -4.245 | 2.19e-05 |
| Undergraduates v. 3^rd^ and 4^th^ Year VS | -0.751 | 0.137 | -5.472 | 4.44e-08 |
| Undergraduates v. Vet Faculty and Staff | -1.188 | 0.127 | -9.362 | 2.22e-16 |
| 1^st^ and 2^nd^ Year VS v. 3rd and 4th VS | -0.217 | 0.142 | -1.530 | 0.126 |
| Vet Faculty and Staff v. 1^st^ and 2^nd^ Year VS | -0.653 | 0.132 | -4.961 | 7.03e-07 |
| Vet Faculty and Staff v. 3^rd^ and 4^th^ Year VS | -0.437 | 0.143 | -3.061 | 0.002 |

**Supplementary Table S14. Trust in a park– General public comparisons.** Participant population group comparisons between the general public and each academic participant population for trust in a park ratings using a linear mixed effects model with fixed effects for participant population and random effects for the individual. Estimate represents the estimated average difference in rating for the academic participant population of interest compared to the general public. Negative estimates indicate that the academic participant populations rated dogs lower (less trust) compared to the general public participant population. Positive estimates indicate that the academic participant populations rated dogs higher (more trust) compared to the general public participant population. The following abbreviations were used: VS = student and vet = veterinary.

| Population Comparison | Estimate | Std. Error | z-statistic | p-value |
| --- | --- | --- | --- | --- |
| General public v. Undergraduates | 0.362 | 0.105 | 3.441 | 5.79e-4 |
| General public v. 1st and 2nd Year VS | -0.310 | 0.112 | -2.772 | 0.006 |
| General public v. 3rd and 4th Year VS | -0.458 | 0.126 | -3.635 | 2.78e-4 |
| General public v. Vet Faculty and Staff | -1.077 | 0.113 | -9.516 | 2.22e-16 |

**Supplementary Table S15. Trust in a park - Academic population comparisons.** Academic participant population comparisons for trust in a park ratings using a linear mixed effects model with fixed effects for participant population and random effects for the individual. Estimates were obtained using linear contrasts. Estimate represents the estimated average difference in trust rating for the participant population with more training compared to the participant population with less training. Negative estimates indicate that the participant population with more training rated dogs lower (less trust) compared to the participant population with less training. Positive estimates indicate that the participant population with more training rated dogs higher (more trust) compared to the participant population with more training. The following abbreviations were used: VS = student and vet = veterinary.

| Population Comparison | Estimate | Std. Error | z-statistic | *p*-value |
| --- | --- | --- | --- | --- |
| Undergraduates v. 1^st^ and 2^nd^ Year VS | -0.673 | 0.133 | -5.041 | 4.64e-07 |
| Undergraduates v. 3^rd^ and 4^th^ Year VS | -0.821 | 0.146 | -5.639 | 1.71e-08 |
| Undergraduates v. Vet Faculty and Staff | -1.439 | 0.135 | -10.699 | 2.22e-16 |
| 1^st^ and 2^nd^ Year VS v. 3rd and 4th VS | -0.148 | 0.150 | -0.985 | 0.325 |
| Vet Faculty and Staff v. 1^st^ and 2^nd^ Year VS | -0.767 | 0.140 | -5.488 | 4.06e-08 |
| Vet Faculty and Staff v. 3^rd^ and 4^th^ Year VS | -0.619 | 0.151 | -4.089 | 4.33e-05 |

**Supplementary Table 16. Feelings thermometer and likelihood of adoption– General public comparisons.** Participant population comparisons between the general public and academic participant populations for the estimated relationship between likelihood of adoption ratings and feelings thermometer ratings using a linear mixed effects model with fixed effects for feelings thermometer, participant population, and their interaction, as well as random effects for the individual. Estimate is the difference in slope for the academic participant populations relative to the general public. Negative estimates indicate that the academic participant populations had a more negative relationship between likelihood of adoption ratings and feelings thermometer ratings compared to the general public participant population. Positive estimates indicate that the academic participant population had a more positive relationship between likelihood of adoption ratings and feelings thermometer ratings compared to the general public participant population. The following abbreviations were used: VS = student and vet = veterinary.

| Population Comparison | Estimate | Std. Error | z-statistic | p-value |
| --- | --- | --- | --- | --- |
| General public v. Undergraduates | 0.034 | 0.001 | 23.075 | 2.22e-16 |
| General public v. 1st and 2nd Year VS | 0.041 | 0.002 | 26.687 | 2.22e-16 |
| General public v. 3rd and 4th Year VS | 0.043 | 0.002 | 25.669 | 2.22e-16 |
| General Public v. Vet Faculty and Staff | 0.036 | 0.002 | 23.497 | 2.22e-16 |

**Supplementary Table 17. Feelings thermometer and likelihood of adoption - Academic population comparisons.** Academic participant population comparisons for the estimated relationship between likelihood to adopt ratings and feelings thermometer ratings using a linear mixed effects model with fixed effects for feelings thermometer, participant population, and their interaction, as well as random effects for the individual. Estimates are obtained using linear contrasts. Estimate is the difference in slope for the participant population with more training compared to the participant population with less training. Negative estimates indicate that the academic participant population with more training had a more negative relationship between likelihood to adopt ratings and feelings thermometer ratings compared to the participant population with less training. Positive estimates indicate that the participant population with more training had a more positive relationship between likelihood to adopt ratings and feelings thermometer ratings compared to the participant population with less training. The following abbreviations were used: VS = student and vet = veterinary.

| Population Comparison | Estimate | Std. Error | z-statistic | *p*-value |
| --- | --- | --- | --- | --- |
| Undergraduates v. 1^st^ and 2^nd^ Year VS | 0.007 | 0.002 | 4.026 | 5.66e-05 |
| Undergraduates v. 3^rd^ and 4^th^ Year VS | 0.009 | 0.002 | 4.941 | 7.79e-07 |
| Undergraduates v. Vet Faculty and Staff | 0.002 | 0.002 | 0.952 | 0.341 |
| 1^st^ and 2^nd^ Year VS v. 3^rd^ and 4^th^ Year VS | 0.002 | 0.002 | 1.181 | 0.238 |
| Vet Faculty and Staff v. 1^st^ and 2^nd^ Year VS | -0.005 | 0.002 | -3.017 | 0.003 |
| Vet Faculty and Staff v. 3^rd^ and 4^th^ Year VS | -0.007 | 0.002 | -3.991 | 6.57e-05 |

**Supplementary Table 18. Feelings thermometers and trust with children – General public comparisons.** Participant population comparisons between the general public and academic participant populations for the estimated relationship between trust with children ratings and feelings thermometer ratings using a linear mixed effects model with fixed effects for feelings thermometer, participant population, and their interaction, as well as random effects for the individual. Estimate is the difference in slope for the academic participant populations relative to the general public. Negative estimates indicate that the academic participant populations had a more negative relationship between trust ratings and feelings thermometer ratings compared to the general public participant population. Positive estimates indicate that the academic participant population had a more positive relationship between trust ratings and feelings thermometer ratings compared to the general public participant population. The following abbreviations were used: VS = student and vet = veterinary.

| Population Comparison | Estimate | Std. Error | z-statistic | p-value |
| --- | --- | --- | --- | --- |
| General public v. Undergraduates | 0.009 | 0.002 | 5.726 | 1.03e-08 |
| General public v. 1st and 2nd Year VS | 0.017 | 0.002 | 10.774 | 2.22e-16 |
| General public v. 3rd and 4th Year VS | 0.017 | 0.002 | 9.798 | 2.22e-16 |
| General Public v. Vet Faculty and Staff | 0.016 | 0.002 | 10.019 | 2.22e-16 |

**Supplementary Table 19. Feelings thermometers and trust with children - Academic population comparisons**. Academic participant population comparisons for the estimated relationship between trust ratings and feelings thermometer ratings using a linear mixed effects model with fixed effects for feelings thermometer, participant population, and their interaction, as well as random effects for the individual. Estimates are obtained using linear contrasts. Estimate is the difference in slope for the participant population with more training compared to the participant population with less training. Negative estimates indicate that the academic participant population with more training had a more negative relationship between trust ratings and feelings thermometer ratings compared to the participant population with less training. Positive estimates indicate that the participant population with more training had a more positive relationship between trust ratings and feelings thermometer ratings compared to the participant population with less training. The following abbreviations were used: VS = student and vet = veterinary.

| Population Comparison | Estimate | Std. Error | z-statistic | *p*-value |
| --- | --- | --- | --- | --- |
| Undergraduates v. 1^st^ and 2^nd^ Year VS | 0.008 | 0.002 | 4.758 | 1.95e-06 |
| Undergraduates v. 3^rd^ and 4^th^ Year VS | 0.008 | 0.002 | 4.378 | 1.20e-05 |
| Undergraduates v. Vet Faculty and Staff | 0.007 | 0.002 | 4.021 | 5.80e-05 |
| 1^st^ and 2^nd^ Year VS v. 3^rd^ and 4^th^ Year VS | 0.000 | 0.002 | -0.030 | 0.976 |
| Vet Faculty and Staff v. 1^st^ and 2^nd^ Year VS | -0.001 | 0.002 | -0.752 | 0.452 |
| Vet Faculty and Staff v. 3^rd^ and 4^th^ Year VS | -0.001 | 0.002 | -0.668 | 0.504 |

**Supplementary Table 20. Feelings thermometer and trust with a cat – General public comparisons.** Participant population comparisons between the general public and academic participant populations for the estimated relationship between trust with a cat ratings and feelings thermometer ratings using a linear mixed effects model with fixed effects for feelings thermometer, participant population, and their interaction, as well as random effects for the individual. Estimate is the difference in slope for the academic participant populations relative to the general public. Negative estimates indicate that the academic participant populations had a more negative relationship between likelihood of adoption ratings and feelings thermometer ratings compared to the general public participant population. Positive estimates indicate that the academic participant population had a more positive relationship between likelihood of adoption ratings and feelings thermometer ratings compared to the general public participant population. The following abbreviations were used: VS = student and vet = veterinary.

| Population Comparison | Estimate | Std. Error | z-statistic | p-value |
| --- | --- | --- | --- | --- |
| General public v. Undergraduates | -0.001 | 0.002 | -0.749 | 0.454 |
| General public v. 1st and 2nd Year VS | 0.001 | 0.002 | 0.515 | 0.607 |
| General public v. 3rd and 4th Year VS | -0.006 | 0.002 | -2.943 | 0.003 |
| General Public v. Vet Faculty and Staff | 0.005 | 0.002 | 3.064 | 0.002 |

**Supplementary Table 21. Feelings thermometer and trust with a cat - Academic population comparisons.** Academic participant population comparisons for the estimated relationship between trust with a cat ratings and feelings thermometer ratings using a linear mixed effects model with fixed effects for feelings thermometer, participant population, and their interaction, as well as random effects for the individual. Estimates are obtained using linear contrasts. Estimate is the difference in slope for the participant population with more training compared to the participant population with less training. Negative estimates indicate that the academic participant population with more training had a more negative relationship between trust ratings and feelings thermometer ratings compared to the participant population with less training. Positive estimates indicate that the participant population with more training had a more positive relationship between trust ratings and feelings thermometer ratings compared to the participant population with less training. The following abbreviations were used: VS = student and vet = veterinary.

| Population Comparison | Estimate | Std. Error | z-statistic | *p*-value |
| --- | --- | --- | --- | --- |
| Undergraduates v. 1^st^ and 2^nd^ Year VS | 0.002 | 0.002 | 1.119 | 0.263 |
| Undergraduates v. 3^rd^ and 4^th^ Year VS | -0.004 | 0.002 | -2.099 | 0.036 |
| Undergraduates v. Vet Faculty and Staff | 0.006 | 0.002 | 3.426 | 6.12e-4 |
| 1^st^ and 2^nd^ Year VS v. 3^rd^ and 4^th^ Year VS | -0.006 | 0.002 | -3.061 | 0.002 |
| Vet Faculty and Staff v. 1^st^ and 2^nd^ Year VS | 0.004 | 0.002 | 2.234 | 0.025 |
| Vet Faculty and Staff v. 3^rd^ and 4^th^ Year VS | 0.011 | 0.002 | 5.155 | 2.53e-07 |

**Supplementary Table 22. Feelings thermometer and trust in a crowd – General public comparisons.** Participant population comparisons between the general public and academic participant populations for the estimated relationship between trust in a crowd ratings and feelings thermometer ratings using a linear mixed effects model with fixed effects for feelings thermometer, participant population, and their interaction, as well as random effects for the individual. Estimate is the difference in slope for the academic participant populations relative to the general public. Negative estimates indicate that the academic participant populations had a more negative relationship between likelihood of adoption ratings and feelings thermometer ratings compared to the general public participant population. Positive estimates indicate that the academic participant population had a more positive relationship between likelihood of adoption ratings and feelings thermometer ratings compared to the general public participant population. The following abbreviations were used: VS = student and vet = veterinary.

| Population Comparison | Estimate | Std. Error | z-statistic | p-value |
| --- | --- | --- | --- | --- |
| General public v. Undergraduates | 0.007 | 0.001 | 4.456 | 8.35e-06 |
| General public v. 1st and 2nd Year VS | 0.009 | 0.002 | 5.839 | 5.26e-09 |
| General public v. 3rd and 4th Year VS | 0.008 | 0.002 | 4.914 | 8.91e-07 |
| General Public v. Vet Faculty and Staff | 0.011 | 0.002 | 7.363 | 1.79e-13 |

**Supplementary Table 23. Feelings thermometer and trust in a crowd - Academic population comparisons.** Academic participant population comparisons for the estimated relationship between trust in a crowd ratings and feelings thermometer ratings using a linear mixed effects model with fixed effects for feelings thermometer, participant population, and their interaction, as well as random effects for the individual. Estimates are obtained using linear contrasts. Estimate is the difference in slope for the participant population with more training compared to the participant population with less training. Negative estimates indicate that the academic participant population with more training had a more negative relationship between trust ratings and feelings thermometer ratings compared to the participant population with less training. Positive estimates indicate that the participant population with more training had a more positive relationship between trust ratings and feelings thermometer ratings compared to the participant population with less training. The following abbreviations were used: VS = student and vet = veterinary.

| Population Comparison | Estimate | Std. Error | z-statistic | *p*-value |
| --- | --- | --- | --- | --- |
| Undergraduates v. 1^st^ and 2^nd^ Year VS | 0.002 | 0.002 | 1.398 | 0.162 |
| Undergraduates v. 3^rd^ and 4^th^ Year VS | 0.002 | 0.002 | 0.916 | 0.360 |
| Undergraduates v. Vet Faculty and Staff | 0.005 | 0.002 | 2.738 | 0.006 |
| 1^st^ and 2^nd^ Year VS v. 3^rd^ and 4^th^ Year VS | -0.001 | 0.002 | -0.370 | 0.712 |
| Vet Faculty and Staff v. 1^st^ and 2^nd^ Year VS | 0.002 | 0.002 | 1.292 | 0.196 |
| Vet Faculty and Staff v. 3^rd^ and 4^th^ Year VS | 0.003 | 0.002 | 1.572 | 0.116 |

**Supplementary Table 24. Feelings thermometer and trust in a park – General public comparisons.** Participant population comparisons between the general public and academic participant populations for the estimated relationship between trust in a park ratings and feelings thermometer ratings using a linear mixed effects model with fixed effects for feelings thermometer, participant population, and their interaction, as well as random effects for the individual. Estimate is the difference in slope for the academic participant populations relative to the general public. Negative estimates indicate that the academic participant populations had a more negative relationship between likelihood of adoption ratings and feelings thermometer ratings compared to the general public participant population. Positive estimates indicate that the academic participant population had a more positive relationship between likelihood of adoption ratings and feelings thermometer ratings compared to the general public participant population. The following abbreviations were used: VS = student and vet = veterinary.

| Population Comparison | Estimate | Std. Error | z-statistic | *p*-value |
| --- | --- | --- | --- | --- |
| General public v. Undergraduates | 0.009 | 0.001 | 6.510 | 7.52e-11 |
| General public v. 1^st^ and 2^nd^ Year VS | 0.016 | 0.002 | 10.690 | 2.22e-16 |
| General public v. 3^rd^ and 4^th^ Year VS | 0.014 | 0.002 | 8.558 | 2.22e-16 |
| General Public v. Vet Faculty and Staff | 0.012 | 0.001 | 8.313 | 2.22e-16 |

**Supplementary Table 25. Feelings thermometer and trust in a park - Academic population comparisons.** Academic participant population comparisons for the estimated relationship between trust in a park ratings and feelings thermometer ratings using a linear mixed effects model with fixed effects for feelings thermometer, participant population, and their interaction, as well as random effects for the individual. Estimates are obtained using linear contrasts. Estimate is the difference in slope for the participant population with more training compared to the participant population with less training. Negative estimates indicate that the academic participant population with more training had a more negative relationship between trust ratings and feelings thermometer ratings compared to the participant population with less training. Positive estimates indicate that the participant population with more training had a more positive relationship between trust ratings and feelings thermometer ratings compared to the participant population with less training. The following abbreviations were used: VS = student and vet = veterinary.

| Population Comparison | Estimate | Std. Error | z-statistic | *p*-value |
| --- | --- | --- | --- | --- |
| Undergraduates v. 1^st^ and 2^nd^ Year VS | 0.007 | 0.002 | 4.002 | 6.28e-05 |
| Undergraduates v. 3^rd^ and 4^th^ Year VS | 0.005 | 0.002 | 2.604 | 0.009 |
| Undergraduates v. Vet Faculty and Staff | 0.003 | 0.002 | 1.793 | 0.073 |
| 1^st^ and 2^nd^ Year VS v. 3^rd^ and 4^th^ Year VS | -0.002 | 0.002 | -1.079 | 0.281 |
| Vet Faculty and Staff v. 1^st^ and 2^nd^ Year VS | -0.004 | 0.002 | -2.176 | 0.030 |
| Vet Faculty and Staff v. 3^rd^ and 4^th^ Year VS | -0.002 | 0.002 | -0.937 | 0.349 |

**Supplementary Table S26. Clinical experience and Likelihood of adoption.** Likelihood of adoption comparisons by dog breed between undergraduates who have clinical experience and those who do not using a linear regression model. Estimate represents the estimated average difference between populations; negative estimates indicate that the undergraduates with clinical experience rated dogs lower (less likely to adopt) compared to those without clinical experience.

| Breed | Estimate | Std. Error | Degrees of Freedom | t-statistic | p-value |
| --- | --- | --- | --- | --- | --- |
| All | -0.559 | 0.201 | 359 | -2.785 | 0.006 |
| Border collie | -0.299 | 0.271 | 358 | -1.100 | 0.272 |
| Boston terrier | -0.607 | 0.342 | 358 | -1.776 | 0.077 |
| Chihuahua | -0.851 | 0.361 | 357 | -2.355 | 0.019 |
| German shepherd | -0.513 | 0.287 | 358 | -1.788 | 0.075 |
| Golden retriever | 0.028 | 0.226 | 358 | 0.124 | 0.901 |
| Jack Russell terrier | -1.178 | 0.339 | 358 | -3.472 | 5.81e-4 |
| Labrador retriever | 0.167 | 0.223 | 358 | 0.748 | 0.455 |
| Maltese | -1.401 | 0.376 | 358 | -3.731 | 2.216e-4 |
| Pitbull | 0.509 | 0.290 | 357 | 1.757 | 0.080 |
| Siberian husky | -0.727 | 0.317 | 359 | -2.289 | 0.023 |
| Dog 11 (Beagle, Australian shepherd) | -0.563 | 0.266 | 356 | -2.117 | 0.035 |
| Dog 12 (Great Dane, German shepherd) | 0.253 | 0.301 | 356 | 0.841 | 0.401 |
| Dog 13 (Staffordshire terrier (pitbull), trace breeds) | -0.922 | 0.311 | 356 | -2.965 | 0.003 |
| Dog 14 (Poodle – small, Poodle – standard) | -1.281 | 0.333 | 356 | -3.847 | 1.42e-4 |
| Dog 15 (Poodle – small, Shih tzu) | -1.599 | 0.352 | 356 | -4.543 | 7.59e-06 |
| Dog 16 (Staffordshire terrier (pitbull), Boxer) | -0.005 | 0.270 | 356 | -0.018 | 0.986 |

**Supplementary Table S27. Clinical experience and trust with children.** Trust with children comparisons by dog breed between undergraduates who have clinical experience and those who do not using a linear regression model. Estimate represents the estimated average difference between populations; negative estimates indicate that the undergraduates with clinical experience rated dogs lower (less trust) compared to those without clinical experience.

| Breed | Estimate | Std. Error | Degrees of Freedom | t-statistic | p-value |
| --- | --- | --- | --- | --- | --- |
| All | -0.835 | 0.169 | 359 | -4.942 | 1.19e-06 |
| Border collie | -1.198 | 0.255 | 358 | -4.696 | 3.79e-06 |
| Boston terrier | -0.480 | 0.261 | 358 | -1.842 | 0.066 |
| Chihuahua | -1.477 | 0.328 | 357 | -4.501 | 9.16e-06 |
| German shepherd | -0.906 | 0.280 | 358 | -3.239 | 0.001 |
| Golden retriever | -0.236 | 0.171 | 358 | -1.377 | 0.169 |
| Jack Russell terrier | -1.470 | 0.279 | 358 | -5.266 | 2.41e-07 |
| Labrador retriever | -0.032 | 0.196 | 358 | -0.163 | 0.871 |
| Maltese | -1.217 | 0.290 | 358 | -4.190 | 3.52e-05 |
| Pitbull | -0.090 | 0.241 | 357 | -0.372 | 0.710 |
| Siberian husky | -1.092 | 0.261 | 359 | -4.183 | 3.62e-05 |
| Dog 11 (Beagle, Australian shepherd) | -0.767 | 0.221 | 356 | -3.478 | 5.67e-4 |
| Dog 12 (Great Dane, German shepherd) | -0.519 | 0.254 | 356 | -2.047 | 0.041 |
| Dog 13 (Staffordshire terrier (pitbull), trace breeds) | -1.259 | 0.226 | 356 | -5.572 | 4.97e-08 |
| Dog 14 (Poodle – small, Poodle – standard) | -0.849 | 0.221 | 356 | -3.849 | 1.41e-4 |
| Dog 15 (Poodle – small, Shih tzu) | -1.178 | 0.257 | 356 | -4.581 | 6.42e-06 |
| Dog 16 (Staffordshire terrier (pitbull), Boxer) | -0.590 | 0.228 | 356 | -2.587 | 0.010 |

**Supplementary Table S28. Clinical experience and trust with a cat.** Trust with a cat comparisons by dog breed between undergraduates who have clinical experience and those who do not using a linear regression model. Estimate represents the estimated average difference between populations; negative estimates indicate that the undergraduates with clinical experience rated dogs lower (less trust) compared to those without clinical experience.

| Breed | Estimate | Std. Error | Degrees of Freedom | t-statistic | p-value |
| --- | --- | --- | --- | --- | --- |
| All | -0.709 | 0.184 | 359 | -3.850 | 1.40e-4 |
| Border collie | -1.102 | 0.273 | 358 | -4.043 | 6.48e-05 |
| Boston terrier | -0.167 | 0.259 | 358 | -0.645 | 0.519 |
| Chihuahua | -0.900 | 0.305 | 357 | -2.951 | 0.003 |
| German shepherd | -0.763 | 0.287 | 358 | -2.657 | 0.008 |
| Golden retriever | -0.159 | 0.225 | 358 | -0.706 | 0.480 |
| Jack Russell terrier | -1.242 | 0.296 | 358 | -4.203 | 3.33e-05 |
| Labrador retriever | 0.107 | 0.242 | 358 | 0.443 | 0.658 |
| Maltese | -0.581 | 0.277 | 358 | -2.099 | 0.037 |
| Pitbull | -0.143 | 0.273 | 357 | -0.522 | 0.602 |
| Siberian husky | -0.946 | 0.288 | 359 | -3.279 | 0.001 |
| Dog 11 (Beagle, Australian shepherd) | -0.888 | 0.255 | 356 | -3.483 | 5.57e-4 |
| Dog 12 (Great Dane, German shepherd) | -0.642 | 0.264 | 356 | -2.427 | 0.016 |
| Dog 13 (Staffordshire terrier (pitbull), trace breeds) | -1.211 | 0.239 | 356 | -5.061 | 6.70e-07 |
| Dog 14 (Poodle – small, Poodle – standard) | -0.784 | 0.239 | 356 | -3.274 | 0.001 |
| Dog 15 (Poodle – small, Shih tzu) | -1.162 | 0.249 | 356 | -4.675 | 4.19e-06 |
| Dog 16 (Staffordshire terrier (pitbull), Boxer) | -0.841 | 0.238 | 356 | -3.535 | 4.62e-4 |

**Supplementary Table S29. Clinical experience and trust in a crowd.** Trust in a crowd comparisons by dog breed between undergraduates who have clinical experience and those who do not using a linear regression model. Estimate represents the estimated average difference between populations; negative estimates indicate that the undergraduates with clinical experience rated dogs lower (less trust) compared to those without clinical experience.

| Breed | Estimate | Std. Error | Degrees of Freedom | t-statistic | p-value |
| --- | --- | --- | --- | --- | --- |
| All | -0.583 | 0.168 | 359 | -3.479 | 5.65e-4 |
| Border collie | -0.909 | 0.232 | 358 | -3.916 | 1.08e-4 |
| Boston terrier | -0.125 | 0.266 | 358 | -0.471 | 0.638 |
| Chihuahua | -1.107 | 0.324 | 357 | -3.411 | 7.21e-4 |
| German shepherd | -0.874 | 0.265 | 358 | -3.303 | 0.001 |
| Golden retriever | 0.157 | 0.171 | 358 | 0.918 | 0.359 |
| Jack Russell terrier | -0.835 | 0.266 | 358 | -3.138 | 0.002 |
| Labrador retriever | 0.223 | 0.183 | 358 | 1.219 | 0.223 |
| Maltese | -0.775 | 0.278 | 358 | -2.789 | 0.006 |
| Pitbull | -0.239 | 0.256 | 357 | -0.936 | 0.350 |
| Siberian husky | -0.931 | 0.244 | 359 | -3.821 | 1.57 |
| Dog 11 (Beagle, Australian shepherd) | -0.599 | 0.210 | 356 | -2.851 | 0.005 |
| Dog 12 (Great Dane, German shepherd) | -0.340 | 0.222 | 356 | -1.528 | 0.127 |
| Dog 13 (Staffordshire terrier (pitbull), trace breeds) | -0.891 | 0.227 | 356 | -3.917 | 1.07e-4 |
| Dog 14 (Poodle – small, Poodle – standard) | -0.547 | 0.206 | 356 | -2.648 | 0.008 |
| Dog 15 (Poodle – small, Shih tzu) | -0.912 | 0.242 | 356 | -3.772 | 1.90 |
| Dog 16 (Staffordshire terrier (pitbull), Boxer) | -0.626 | 0.222 | 356 | -2.822 | 0.005 |

**Supplementary Table S30. Clinical experience and trust in a park.** Trust in a park comparisons by dog breed between undergraduates who have clinical experience and those who do not using a linear regression model. Estimate represents the estimated average difference between populations; negative estimates indicate that the undergraduates with clinical experience rated dogs lower (less trust) compared to those without clinical experience.

| Breed | Estimate | Std. Error | Degrees of Freedom | t-statistic | p-value |
| --- | --- | --- | --- | --- | --- |
| All | -0.615 | 0.162 | 359 | -3.785 | 1.80 |
| Border collie | -0.705 | 0.200 | 358 | -3.527 | 4.74e-4 |
| Boston terrier | -0.403 | 0.252 | 358 | -1.604 | 0.110 |
| Chihuahua | -1.123 | 0.341 | 357 | -3.289 | 0.001 |
| German shepherd | -0.825 | 0.243 | 358 | -3.399 | 7.53e-4 |
| Golden retriever | 0.073 | 0.147 | 358 | 0.495 | 0.621 |
| Jack Russell terrier | -0.696 | 0.247 | 358 | -2.816 | 0.005 |
| Labrador retriever | -0.019 | 0.152 | 358 | -0.124 | 0.902 |
| Maltese | -0.842 | 0.314 | 358 | -2.681 | 0.008 |
| Pitbull | -0.404 | 0.249 | 357 | -1.623 | 0.105 |
| Siberian husky | -0.969 | 0.234 | 359 | -4.149 | 4.17e-05 |
| Dog 11 (Beagle, Australian shepherd) | -0.589 | 0.204 | 356 | -2.892 | 0.004 |
| Dog 12 (Great Dane, German shepherd) | -0.494 | 0.211 | 356 | -2.344 | 0.0196 |
| Dog 13 (Staffordshire terrier (pitbull), trace breeds) | -0.842 | 0.227 | 356 | -3.709 | 2.41e-4 |
| Dog 14 (Poodle – small, Poodle – standard) | -0.500 | 0.189 | 356 | -2.645 | 0.009 |
| Dog 15 (Poodle – small, Shih tzu) | -0.819 | 0.243 | 356 | -3.374 | 8.22e-4 |
| Dog 16 (Staffordshire terrier (pitbull), Boxer) | -0.737 | 0.207 | 356 | -3.562 | 4.18e-4 |
